# Folding of Tubular Waterbomb

**DOI:** 10.34133/2020/1735081

**Published:** 2020-04-10

**Authors:** Jiayao Ma, Huijuan Feng, Yan Chen, Degao Hou, Zhong You

**Affiliations:** ^1^Key Laboratory of Mechanism Theory and Equipment Design of Ministry of Education, Tianjin University, 135 Yaguan Road, Tianjin 300350, China; ^2^School of Mechanical Engineering, Tianjin University, 135 Yaguan Road, Tianjin 300350, China; ^3^Department of Engineering Science, University of Oxford, Parks Road, Oxford OX1 3PJ, UK

## Abstract

Origami has recently emerged as a promising building block of mechanical metamaterials because it offers a purely geometric design approach independent of scale and constituent material. The folding mechanics of origami-inspired metamaterials, i.e., whether the deformation involves only rotation of crease lines (rigid origami) or both crease rotation and facet distortion (nonrigid origami), is critical for fine-tuning their mechanical properties yet very difficult to determine for origami patterns with complex behaviors. Here, we characterize the folding of tubular waterbomb using a combined kinematic and structural analysis. We for the first time uncover that a waterbomb tube can undergo a mixed mode involving both rigid origami motion and nonrigid structural deformation, and the transition between them can lead to a substantial change in the stiffness. Furthermore, we derive theoretically the range of geometric parameters for the transition to occur, which paves the road to program the mechanical properties of the waterbomb pattern. We expect that such analysis and design approach will be applicable to more general origami patterns to create innovative programmable metamaterials, serving for a wide range of applications including aerospace systems, soft robotics, morphing structures, and medical devices.

## 1. Introduction

Mechanical metamaterials are artificially designed structures that offer extreme and unusual, yet useful, mechanical properties determined by their structural and geometric configurations rather than only intrinsic material properties of their composing elements. Conventional mechanical metamaterials are often formed by quasi-1D rods or links [1–6]. Recently, origami has emerged as a promising building block of mechanical metamaterials with versatile functionalities and programmability [7–17], due to its capability of transforming a 2D crease pattern into a complex 3D sculpture, purely geometric traits independent of both scale and constituent materials, and ease of manufacturing [18, 19].

The mechanical properties of an origami metamaterial are primarily determined by its folding mechanics. When rigid patterns such as Miura-ori [7, 8] are utilized, the facets do not stretch or bend but only rotate about the creases, and the metamaterials behave like a kinematic mechanism during folding. On the other hand, nonrigid ones [15, 16] enable simultaneous crease rotation and facet distortion, resulting in structural deformation of the metamaterials. Difference in folding mechanics leads to distinct mechanical properties such as stiffness [15]. Here, we report a tubular waterbomb pattern with a transition between rigid origami motion and structural deformation, making it possible for programming the behavior of the derived metamaterial.

In origami, the waterbomb tube refers to an origami structure made from a crease pattern obtained by tessellation of the waterbomb bases. A typical waterbomb base is a six-crease pattern with two colinear mountain creases and four diagonal valley ones intersecting at a common vertex [20]. The base has been used to create many fascinating origami objects including the structure that is the focus of this article [21–23]. One of the most distinctive characteristics of the waterbomb tube is that it has a negative Poisson's ratio: when compressed, both its length and radius get smaller. This has led to a number of notable practical applications such as an expandable medical stent graft [24], a transformable worm robot [25], and a deformable robot wheel [26]. Recently, the authors also obtained programmable stiffness and shape modulation in the waterbomb tube using a bar-and-hinge numerical model [27]. Despite that, its precise folding behaviors and mechanical properties have remained ambiguous. Therefore, in this paper, we aim to expose the exact folding mechanics of the waterbomb tube by means of kinematics and structural analysis.

## 2. Kinematic Modelling of Rigid Folding

**Figure 1:**
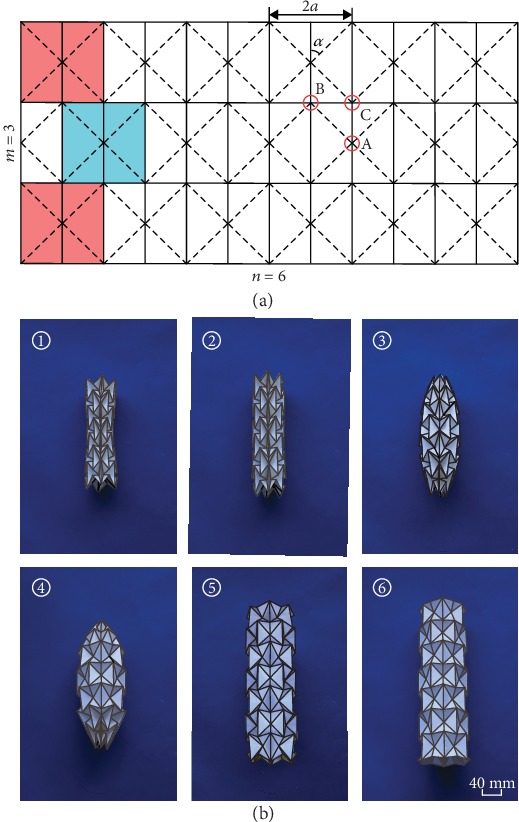
The waterbomb origami pattern and model. (a) The waterbomb pattern formed by tessellating the waterbomb bases. Solid and dashed lines represent mountain and valley creases, respectively. A typical base is shown in blue, which is placed side by side forming the middle row. On the adjacent rows, the bases are shifted by half a base (red). Four geometric parameters: width of the base 2*a*, sector angle *α*, the numbers of bases in the longitudinal direction *m*, and circumferential direction *n*—completely defining the pattern. A, B, and C are the three groups of representative vertices. (b) Deployment of a card model of a waterbomb tube. The model was made from conventional cards of 0.3 mm in thickness obtained from stationery stores. The geometric parameters were 2*a* = 46 mm, *α* = 45°, *m* = 7, and *n* = 6.

Figure 1(a) illustrates the crease pattern of a waterbomb tube defined by four independent geometric parameters—width 2*a*, sector angle *α*, and the number of bases longitudinally, *m*, and circumferentially, *n*. When we join together the left and right edges of the pattern, we can obtain a waterbomb tube [21, 22]. We will illustrate the motion behavior of the waterbomb tube with a representative model. First, we create a waterbomb tube in the fully contracted configuration (Figure 1(b) ①), where the facets in the middle row collide. When we slightly expand the tube along its central axis, its radius increases as well as the length, and a uniform radius along the tube is obtained (we shall demonstrate later that such a configuration always exists) (Figure 1(b) ②). With further expansion, it develops a pineapple shape with closure at both ends (Figure 1(b) ③ and ④). Subsequently, it opens up its ends again (Figure 1(b) ⑤) and then regains a uniform radius (Figure 1(b) ⑥). After this, the tube can be only marginally deployed, and the change in shape is hardly noticeable.

**Figure 2:**
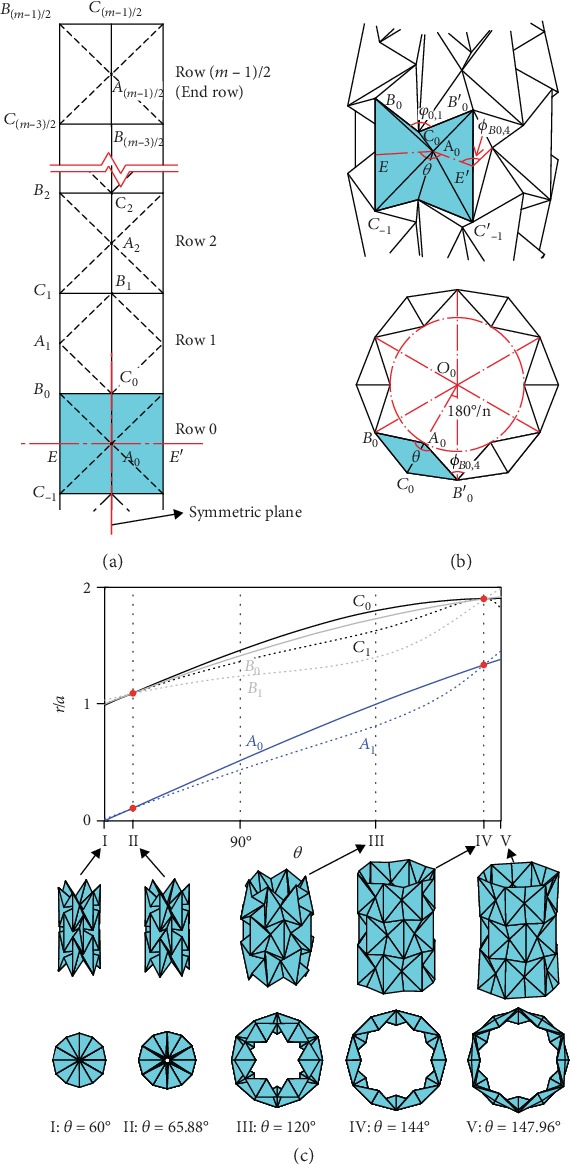
Rigid foldability of the waterbomb tube with an odd *m*. (a) Top half of a longitudinal strip in a waterbomb tube. *E*‐*E*′ is the equator of the tube. Vertices above the equator are marked as *A*_*i*_, *B*_*i*_, and *C*_*i*_, while those below are marked as *A*_‐*i*_, *B*_‐*i*_, and *C*_‐*i*_, in which 0 ≤ *i* ≤ (*m*‐1)/2. (b) The front view of a waterbomb tube with the equatorial row 0 and rows immediately adjacent to it and the top view of the equatorial row. One of the bases on the equatorial row is shown in blue. *O*_0_ is the center of the tube. (c) Variation of nondimensional radii *r*/*a* of vertices *A*_*i*_, *B*_*i*_, and C_*i*_ (*i* = 0, 1) of a tube with *m* = 3, *n* = 6, and *α* = 45° with respect to the folding angle *θ* and five representative configurations I to V of the tube in front and top views. The corresponding angles *θ* are listed below the configurations. Also see SM Movie S1.

To determine the folding mechanics, we first build a kinematic model for the waterbomb tube. The pattern has three distinct vertex groups: central vertex *A* and edge vertices *B* and *C*, the motion of which can be modeled as a spherical 6*R* linkage with three degrees of freedom (DOFs) [28, 29]. As such, the tube is a network of these linkages, leading to a multi-DOF system (details in the supplementary material, S2). Thus, we reduce the DOF by making the following assumptions of symmetry based on our observation in Figure 1(b). First, the motion of the tube is symmetric about the equatorial plane that passes through the middle of the tube and divides it into two identical top and bottom halves. Second, all bases that are circumferentially placed in the same row have identical folding behavior. Finally, each base moves in a plane-symmetric way about a plane (presented as the red dot-dash line in Figure 2(a)) passing through the two mountain creases and the central axis of the tube. We first discuss the case when *m* (i.e., the number of rows in the tube) is odd. Figure 2(a) presents a strip out of the origami pattern forming a tube with an odd number of rows. The equatorial plane passes through the center of the middle row defined as row 0. According to the above assumptions, linkage **A**_0_ is symmetric about the equatorial plane and the plane passing vertex **A**_0_ and tube axis, which reduces its DOF to one. Subsequently, the overall DOF of the tube becomes one, and its motion, described by the respective dihedral angles of all the linkages, can be found out through kinematic analysis (details in the supplementary material, S3).

Consider a particular example with *m* = 3, *n* = 6, and *α* = 45°. As shown in Figure 2(b), *φ*_0,1_ is defined as the dihedral angle between two triangular facets that pass the top mountain crease in linkage **A**_0_. Taking *φ*_0,1_ as the input, we can obtain the other five dihedral angles of the linkage by kinematic analysis. Subsequently, we can determine all dihedral angles in the tube by using these known dihedral angles as inputs for adjacent linkages. To depict the extent of deployment of the tube, we define *θ* as the folding angle between the two largest triangular facets of a base on row 0 and the following equation holds: *θ* = *φ*_0,1_. The nondimensional radii of the vertices, *r*/*a*, are plotted against *θ* in Figure 2(c), together with five representative configurations of the tube during deployment. We can draw three conclusions from the result. First, there exist two particular configurations, II with *θ* = 65.88° and IV with *θ* = 144°. On those configurations, the radii of all the vertices *B*_*i*_ and *C*_*i*_ become equal and so do those of vertices *A*_*i*_ (*i* = 0, 1), see the red dots on the curves. In other words, all the bases take the same geometric form at either configuration, resulting in a tube of a uniform radius. Thus, we prove theoretically that it is possible to construct a uniform waterbomb tube out of rigid sheet materials, provided that the amount of prefolding is correct. Second, the rigid-foldable range of the tube is bounded by two values of *θ*, *θ*_min_ and *θ*_max_. The lower bound *θ*_min_ = 60° corresponds to the compactly folded configuration I: *ϕ*_*B*0,4_ = 0, i.e., two triangular facets on both sides of the common crease *B*_0_*C*_‐1_ overlap entirely, whereas the upper bound *θ*_max_ = 147.96° is associated with the most expanded configuration V in which the upper sides of the bases on row 1 form a regular hexagon with a side length 2*a*. The supplementary material (S3.B. 1) provides detailed derivations on how both bounds are obtained. Third, the uniform radius and the bound configurations divide the tube deployment process with distinct shapes, i.e., a pineapple shape with the largest radius attained at crease *B*_0_*C*_‐1_ in row 0, such as configuration III with *θ* = 120°, when 65.88° < *θ* < 144°, and a dogbone shape with the smallest radius reached at crease *B*_0_*C*_‐1_, when 60° ≤ *θ* < 65.88° and 144° < *θ* ≤ 147.96°.

## 3. Mechanism-Structure-Mechanism Transition

**Figure 3:**
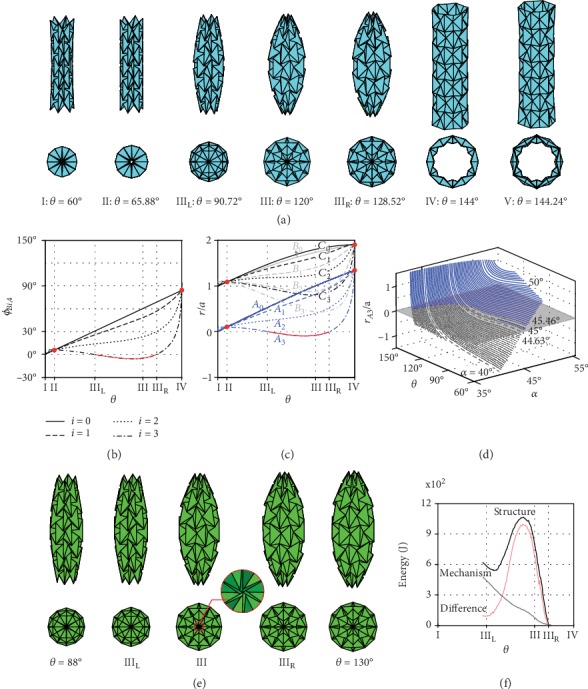
Mechanism-structure-mechanism transition of longer tubes with an odd *m*. (a) Front and top views of the tube with *m* = 7, *n* = 6, and *α* = 45° deploying from configurations I to V. The corresponding folding angles *θ* are listed below the deployment sequence. The tube is completely concealed between III_L_ and III_R_. Also see SM Movie S2. (b) Variation of dihedral angles *ϕ*_*Bi*,4_*vs*. *θ*. The red curve shows that *ϕ*_*B*3,4_ < 0 between III_L_ and III_R_. (c) The nondimensional radii *r*/*a* of vertices *A*_*i*_, *B*_*i*_, and *C*_*i*_ (*i* = 0, 1, 2, 3) *vs.θ*, in which the red curve shows *r*_*A*3_ < 0 between III_L_ and III_R_. (d) Relationship among nondimensional radius of vertices *A*_3_(*r*_*A*3_/*a*), *θ*, and *α*. Some values of *α* are listed alongside their corresponding curves. The shaded plane is where *r*_*A*3_ = 0. Blue solid lines are for *r*_*A*3_ > 0 and grey dashed lines for *r*_*A*3_ < 0 (where physical interference happens). (e) Front and top views of the waterbomb tube undergoing structural deformation. At configurations III_R_ to III_L_, the tube forms a concealed volume. Any in-between configuration, e.g., configuration III, requires structural deformation, as presented in the close up figure where the vertices are squeezed and distorted by each other. (f) Elastic strain energy *vs*. *θ* when the tube is modeled as a mechanism (in grey) and as a structure (in black) and their difference (in red).

When we increase the number of rows, existing rows will retain their motion in the original tube with *m* = 3 and drive concurrently the motion of newly added ones. However, the upper sides of bases on the end rows will form an *n*-sided regular polygon earlier, resulting in the termination of the motion. For example, if we increase *m* to 7, *θ*_min_ remains to be 60° while *θ*_max_ decreases to 144.24°.  Figure 3(a) presents the motion sequence of the tube, where configurations I and V correspond to the minimum and maximum values of *θ*, respectively.  Figure 3(b) plots the dihedral angles *ϕ*_*Bi*,4_ between adjacent bases of row *i* (*i* = 0, 1, 2, 3) against *θ*.  Figure 3(c) gives the radii of the vertices *A*_*i*_, *B*_*i*_, and *C*_*i*_, *r*_*Ai*_, *r*_*Bi*_, and *r*_*Ci*_, *vs.θ*. As in the case of *m* = 3, the curves intersect at two points (marked by red dots), which indicates that the tube also has a uniform radius at configurations II and IV when *m* = 7. Comparing it to the tube with *m* = 3, it is found that the values of *θ* at these two configurations are exactly the same. In fact, it can be proven kinematically that the uniform radius configurations are independent of the number of rows (supplementary material, S3.B.2). We can intuitively imagine that when all bases in the tube are in identical shape, more rows can be added to the tube in a geometrically compatible way.

Furthermore, the tube undergoes nonrigid folding within a region of *θ* between *θ* = 90.72° at configuration III_L_ and *θ* = 128.52° at III_R_ (supplementary material, S3.B.3). This is clearly demonstrated by the intermediate configuration III in Figure 3(a), where the central vertices *A*_3_ of the bases on the two end rows collide with each other. The reason is that the dihedral angle *ϕ*_*B*3,4_ < 0 in this range, indicates interference among the facets on row 3, which is not permitted in rigid origami. This conclusion is further supported by the fact that *r*_A3_ < 0 in the same range as shown in Figure 3(c). Therefore, the tube has rigid origami motion only within two distinct regions of *θ*: 60° ≤ *θ* ≤ 90.72° and 128.52° ≤ *θ* ≤ 144.24°. At configurations III_L_ and III_R_, the ends of the cylinder are closed, and the tube becomes a concealed volume. It has been shown that structural deformation is required for any change in a concealed volume [30]. Thus, in order to move a physical tube specimen from one rigid-foldable range to the other, the component sheet material has to deform, and the tube works as a structure instead of a mechanism. This kind of folding behavior is referred to as *mechanism-structure-mechanism transition*.

The mechanism-structure-mechanism transition leads to a dramatic variation in stiffness for waterbomb-based structures and metamaterials. Within the rigid origami regime, the tube has a low stiffness determined by the torsional stiffness of the creases. When entering the structural range, the stiffness will be significantly increased due to facet deformation taken place in the tube for its shape change. This feature is demonstrated through a structural analysis of a tube using the finite element method. The model had identical *n* = 6, *m* = 7, and *α* = 45° with that in Figure 3(a), and the facets were set to be 2 orders stiffer than the creases to distinguish the deformation of these two components [31–33] (supplementary material, S4). The simulation started from *θ* = 130° near configurations III_R_ and terminated at *θ* = 88° just beyond configuration III_L_. The folding process of the tube is shown in Figure 3(e), from which physical contact and deformation of the facets at the ends are clearly seen at configuration III. The elastic strain energy of the tube is plotted in Figure 3(f). To manifest the effect of facet deformation, a fictional mechanism motion was also simulated for the tube by allowing the facets to freely penetrate into each other. In the mechanism mode, most energy is stored in the creases and distributed almost linearly. In the structural mode, both facets and creases deform. The energy difference between the two acts as an indicator of the level of deviation from rigid origami motion. The larger it is, the more extra deformation is required to enable the tube to move. It is also worth pointing out that the energy difference is not strictly zero at configuration III_L_ where the tube resumes rigid folding. This is due to some localized residual deformation near the vertices in contact in the structural mode, without affecting the global folding behaviour of the tube. Compared with the fictional mechanism mode, a 110% higher maximum strain energy is required to fold the tube through the structural range, indicating a larger stiffness. The precise stiffness variation depends on the sheet material of the tube, but when that is known, it is possible to predict the external loading that will cause the tube to move from one rigid origami range to the other. Notice that the stiffness property of this tubular waterbomb can also be revealed with the bar-and-hinge model such as the Merlin code [31] and the Origami Contact Simulator [34], or the smooth fold model [35].

## 4. Programmability of Folding Mechanics

Using the established kinematic model, we can program the existence and range of the mechanism-structure-mechanism transition by varying geometric parameters of the pattern. Take *m* = 7 and *n* = 6 as an example. The radius of the vertex *A*_3_ and *r*_*A*3_/*avs*. *θ* for various *α* is presented in Figure 3(d), where we can find the transition occurs when 44.63° < *α* < 45.46°. When *α* ≥ 45.46°, the tube conducts a pure rigid origami motion from *θ*_min_ to *θ*_max_ during deployment. Since *θ*_min_ and *θ*_max_ are also related to *α*, the motion range shrinks with *α*. If *α* ≤ 44.63°, the tube experiences only one curtailed rigid origami range due to physical interference. For instance, when *α* = 40°, the rigid motion range is 142.97° ≤ *θ* ≤ 146.54°.

**Figure 4:**
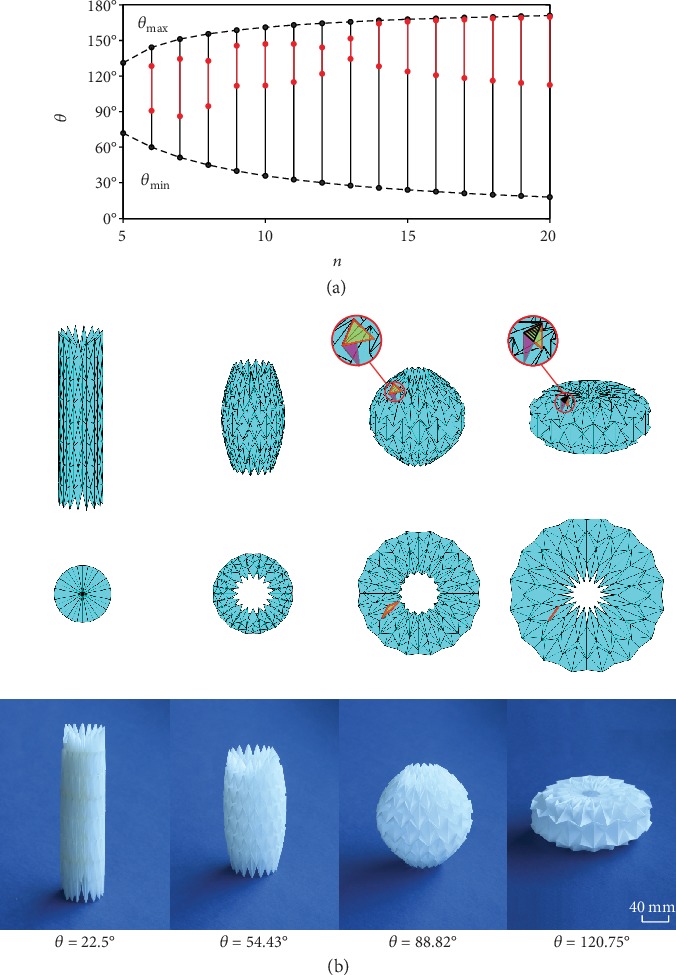
Waterbomb tubes with an odd *m* and varying *n*. (a) Rigid foldable range of tubes with *m* = 7, *α* = 45°, and *n* from 5 to 20. The red lines indicate that the tube is in the structural range. (b) Deployment of a tube with *m* = 7, *α* = 45°, and *n* = 16 and a card model with identical geometry and 2*a* = 46 mm. The corresponding folding angles *θ* are listed below the deployment sequence. The rigid folding range of the tube is 22.5° ≤ *θ* ≤ 120.75°. At the end of rigid folding, the yellow and purple triangular facets hit each other, causing interference in the shaded area. Also see SM Movie S3.

The folding behavior of the waterbomb tube can also be programmed by varying the number of bases in a row. To demonstrate this, consider *m* = 7 and *α* = 45°, but *n* changing from 4 to 20. We find from the theoretical model that rigid folding occurs only when *n* ≥ 5. The upper and lower limits, *θ*_min_ and *θ*_max_, are plotted against *n* in Figure 4(a), together with the transitional structural range (highlighted by red lines) if it exists. The result indicates that the mechanism-structure-mechanism transition consistently appears when *n* ≥ 6. However, when *n* is relatively large, *n* ≥ 14 in the current geometric setup, the mechanism motion range in the neighborhood of *θ*_max_ becomes very narrow, and practically, the tube can be considered reaching a stable configuration rather than another mechanism motion range. Another interesting phenomenon when *n* is large is that the tube can form a spherical shape that was used as an origami wheel [36] or artificial muscles [37]. The deployment process of such a tube with *m* = 7, *α* = 45°, and *n* = 16 is presented in Figure 4(b). Our analysis shows that when *θ* reaches 120.75°, the two triangles in yellow and purple, respectively, meet and overlap at the shaded area, which ceases the rigid folding process before the tube ends are closed.

So far, we have only discussed the situation of *m* being odd. Similar behavior exists when *m* is even. The equatorial row of the tube no longer exists in this case, and the equatorial plane passes through the midpoints of the creases linking vertices *B*_0_ and *C*_0_. However, the tube remains a single DOF system as the two directly adjacent rows above and below the equatorial plane must behave the same under symmetrical assumptions, and they subsequently drive the motion of the remaining rows. Moreover, the tube also has a pair of uniform radius configurations identical to its odd row counterpart with the same *n* and *α*, albeit *m* differs (supplementary material, S3.C). It means that the uniform radius configurations of the tube are solely decided by parameters *α* and *n*. It is not related to *m*.

## 5. Conclusions

We have uncovered the true folding mechanics of the tubular waterbomb and its dependence on pattern geometric parameters. Through a rigorous kinematic analysis, we have demonstrated that some waterbomb tubes are capable of rigid origami motion, whereas others will experience what we refer to as a mechanism-structure-mechanism transition. And a structural analysis has revealed a significant increase in stiffness when the tube transforms into the structural range. Furthermore, we have derived theoretically the correlation between the occurrence and range of the mechanism-structure-mechanism transition and geometric parameters of the pattern, making accurate programming of the mechanical properties readily achievable. Thus, this work can not only facilitate the development of mechanical metamaterials making use of the intriguing properties of the tubular waterbomb but also provide an analysis framework for novel programmable metamaterials with wide engineering applications such as soft robotics, morphing structures, and medical devices. To adopt our analytical model where symmetry assumption was made in these applications, the key is to maintain the symmetry of the pattern during motion. Considering that the thickness of the sheets in the waterbomb pattern cannot be ignored in physical applications, the analytical model should be adjusted to the thick-panel origami model [23]. The motion symmetry would be satisfied automatically by structural constraints introduced by the panel thickness.

## Supplementary Materials

Supplementary 1 Supplementary Information: derivations, figures, and equations.

Supplementary 2 Movie S1: animation of Figure 2(c).

Supplementary 3 Movie S2: animation of Figure 3(a).

Supplementary 4 Movie S3: animation of Figure 4(b).
