# Supplementary material for: Folding of Tubular Waterbomb
Source: Research (Wash D C). 2020 Apr 10;2020:1735081. doi: 10.34133/2020/1735081 (PMC7171592; doi:10.34133/2020/1735081)
Supplement: Supplementary 1 — Supplementary Information: derivations, figures, and equations. [file 1735081.f1.docx]

**Folding of Tubular Waterbomb**

**Supplementary Materials**

Jiayao Ma^1,2†^, Huijuan Feng^1,2†^, Yan Chen^1,2*^, Degao Hou^1,2^, Zhong You^2,3*^

† These authors contributed equally to this work.

* Correspondence should be addressed to: Yan Chen; [yan_chen@tju.edu.cn](mailto:yan_chen@tju.edu.cn) and Zhong You; [zhong.you@eng.ox.ac.uk](mailto:zhong.you@eng.ox.ac.uk)

**This file includes:**

S1. Nomenclature………………………….…………………………………..……..……2

S2. Spherical linkages consisting of only revolute joints………………………………4

S3. Kinematics of the waterbomb tube …………………………………………………5

A. Kinematics of linkages A, B, C …………………………………………………5

B. Motion of a tube with an odd number of rows ……………………………………7

C. Motion of a tube with an even number of rows ……………………………………19

S4. The mechanism-structure-mechanism transition ……………………………………25

Figures S1 to S10

**Other Supplementary information for this manuscript includes the following:**

Movies:

Movie S1 – Animation of Figure 2(c)

Movie S2 – Animation of Figure 3(a)

Movie S3 – Animation of Figure 4(b)

**S1. Nomenclature**

**Major symbols**

$\mathbf{I}_{3}$ 3 by 3 unit matrix

$i$ row number of a waterbomb pattern

$k$ number for axis of a spherical linkage

$\mathbf{Q}_{k(k+1)}$ 3 by 3 transformation matrix between the coordinate system of link $(k-1)k$ and that of link $k(k+1)$ for spherical linkages

$x_{k}$ coordinate axis perpendicular to $z_{k-1}$ and $z_{k}$

$z_{k}$ axis of creases or revolute joints

**Geometric parameters:**

$a$ half width of the waterbomb base

$\alpha$ angle of the waterbomb base

$m$ the number of waterbomb bases in the longitudinal direction of a waterbomb pattern

$n$ the number of waterbomb bases in the circumferential direction of a waterbomb pattern

$\theta$ folding angle between the two largest triangular facets of a waterbomb base on the equatorial row of a waterbomb tube when *m* is odd.

$L$ length of a waterbomb tube

$t$ wall thickness of a waterbomb model and the 3D printed waterbomb tube prototype

$\text{A}_{i}$ and $\text{A}'_{i}$ the central vertices of waterbomb bases on row *i*. $\text{A}'_{i}$ is immediately adjacent to $\text{A}_{i}$

$\text{B}_{i}$ and $\text{B}'_{i}$ the corner vertices of waterbomb bases on row *i*. $\text{B}'_{i}$ is immediately adjacent to $\text{B}_{i}$

$\text{C}_{i}$ and $\text{C}'_{i}$ the middle vertices at the edges of waterbomb bases on row *i*, $\text{C}'_{i}$ is immediately adjacent to $\text{C}_{i}$

$\text{A}_{i}^{\text{p}}$ and $\text{A}'_{i}^{\text{p}}$ projection of vertices $\text{A}_{i}$ and $\text{A}'_{i}$ on the equatorial plane

$\text{B}_{i}^{\text{p}}$ and $\text{B}'_{i}^{\text{p}}$ projection of vertices $\text{B}_{i}$ and $\text{B}'_{i}$ on the equatorial plane

$\text{C}_{i}^{\text{p}}$ and $\text{C}'_{i}^{\text{p}}$ projection of vertices $\text{C}_{i}$ and $\text{C}'_{i}$ on the equatorial plane

E or $\text{E}'$ the midpoints at the vertical side edges of a waterbomb base on the equatorial row of an odd-row tube

**Kinematic parameters:**

$\alpha_{k(k+1)}$ kinematic twist (angle) between axes $z_{k}$and $z_{k+1}$ about axis $x_{k+1}$.

$\theta_{k}$ angle of rotation from $x_{k}$ to $x_{k+1}$ about axis $z_{k}$, also known as the revolute variable of joint $k$

$\delta_{i,k}$ kinematic revolute angle at *k-*th crease of spherical 6*R* linkage **A***_i_*, based on DH notation, *k* = 1, 2, …, 6

$\omega_{\text{B}i,k}$ kinematic revolute angle at *k-*th crease of spherical 6*R* linkage **B***_i_*, based on DH notation, *k* = 1, 2, …, 6

$\omega_{\text{C}i,k}$ kinematic revolute angle at *k-*th crease of spherical 6*R* linkage **C***_i_*, based on DH notation, *k* = 1, 2, …, 6

dihedral angle at *k-*th crease of spherical 6*R* linkage **A***_i_*, *k* = 1, 2, …, 6

dihedral angle at *k-*th crease of spherical 6*R* linkage **B***_i_*, *k* = 1, 2, …, 6

dihedral angle at *k-*th crease of spherical 6*R* linkage **C***_i_*, *k* = 1, 2, …, 6

Insignificant minor symbols will be explained when they appear.

**S2. Spherical linkages consisting of only revolute joints**

In rigid origami, the motion of facets about a vertex can be modeled as a spherical linkage. It is a linkage where adjacent rigid links are connected by only revolute joints (creases) that meet at the vertex. A spherical linkage can be analyzed using the matrix method with the DH notations introduced by Denavit and Hartenberg [36]. Figure S1 shows a portion of such a linkage. For a spherical linkage consisting of 6 links forming a loop, the closure equations are

$\mathbf{Q}_{12}\mathbf{Q}_{23}\cdot\cdot\cdot\mathbf{Q}_{61}=\mathbf{I}_{3}$, (S1)

where

$\mathbf{Q}_{k(k+1)}=\left[\begin{matrix} \cos\theta_{k} & \sin\theta_{k} & 0 \\ -\cos\alpha_{k(k+1)}\sin\theta_{k} & \cos\alpha_{k(k+1)}\cos\theta_{k} & \sin\alpha_{k(k+1)} \\ \sin\alpha_{k(k+1)}\sin\theta_{k} & -\sin\alpha_{k(k+1)}\cos\theta_{k} & \cos\alpha_{k(k+1)} \end{matrix}\right]$, (S2)

and when *k* + 1 = 7, it is replaced by 1. The transformation matrix has the following property.

$\mathbf{Q}_{(k+1)k}=\mathbf{Q}_{k(k+1)}^{-1}=\left[\begin{matrix} \cos\theta_{k} & -\cos\alpha_{k(k+1)}\sin\theta_{k} & \sin\alpha_{k(k+1)}\sin\theta_{k} \\ \sin\theta_{k} & \cos\alpha_{k(k+1)}\cos\theta_{k} & -\sin\alpha_{k(k+1)}\cos\theta_{k} \\ 0 & \sin\alpha_{k(k+1)} & \cos\alpha_{k(k+1)} \end{matrix}\right]$. (S3)


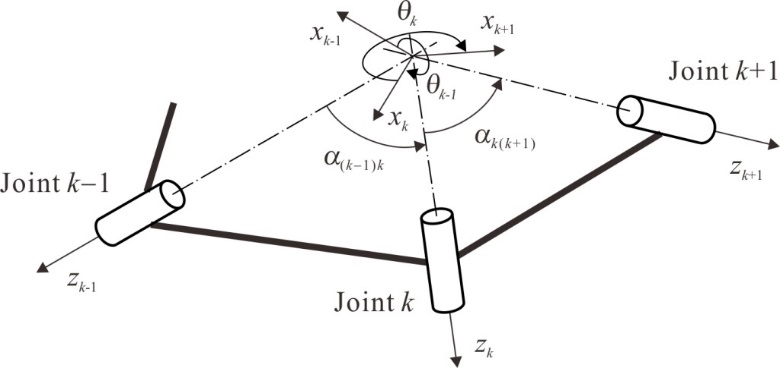


**Figure S1.** A portion of a spherical linkage with only rotational joints. *z_k_* is along the axis of joint *k*. At joint *k*, *x_k_* is perpendicular to axes *z_k_*_-1_ and *z_k_*. *α*(*_k_*_–1)_*_k_* is the angle of rotation from axes *z_k_*_-1_ to *z_k_* positively about axis *x_k_*. *θ* *_k_* is the angle of rotation from *x_k_* to *x_k_*_+1_ positively about *z_k_*.

**S3. Kinematics of the waterbomb tube**

**A. Kinematics of linkages A, B, C**

The waterbomb pattern consists of three distinct spherical 6*R* linkages at vertices A*_i_*, B*_i_* and C*_i_*, Fig. 1(a) in the main text, which are referred to as linkages **A***_i_*, **B***_i_* and **C***_i_* hereafter. All of these linkages carry out a plane-symmetric motion during.

Consider now linkage **A***_i_* (Fig. S2**a**). Its kinematic twists are $\alpha_{12}=\alpha_{34}=\alpha_{45}=\alpha_{61}=\alpha,$ $\alpha_{23}=\alpha_{56} =\pi-2\alpha$. Because of plane-symmetry, there are

$\delta_{i,5}=\delta_{i,3}$, $\delta_{i,6}=\delta_{i,2}$. (S4)

Applying Eq. (S1) to this linkage and considering Eq. (S4), the following equations are obtained

$\text{tan}\frac{\delta_{i,1}}{2}=\frac{\left[\begin{matrix} -\sin\alpha(\cos\alpha\text{sin}\delta_{i,2}\text{sin}\delta_{i,3}+\cos\alpha\cos2\alpha\text{cos}\delta_{i,2}\text{cos}\delta_{i,3}+\sin\alpha\sin2\alpha\text{cos}\delta_{i,3}) \\ +\cos\alpha(\cos\alpha\sin2\alpha\text{cos}\delta_{i,2}-\sin\alpha\cos2\alpha) \end{matrix}\right]}{\sin\alpha(\text{sin}\delta_{i,3}\text{cos}\delta_{i,2}-\cos2\alpha\text{sin}\delta_{i,2}\text{cos}\delta_{i,3})+\cos\alpha\sin2\alpha\text{sin}\delta_{i,2}}$, (S5a)

$\text{tan}\frac{\delta_{i,4}}{2}=\frac{\left[\begin{matrix} \cos\alpha(\cos\alpha\sin2\alpha\text{cos}\delta_{i,3}-\sin\alpha\cos2\alpha\text{cos}\delta_{i,2}\text{cos}\delta_{i,3}-\sin\alpha\text{sin}\delta_{i,2}\text{sin}\delta_{i,3}) \\ -\sin\alpha(\sin\alpha\sin2\alpha\text{cos}\delta_{i,2}+\cos\alpha\cos2\alpha) \end{matrix}\right]}{\sin\alpha(\text{sin}\delta_{i,2}\text{cos}\delta_{i,3}-\cos2\alpha\text{sin}\delta_{i,3}\text{cos}\delta_{i,2})+\cos\alpha\sin2\alpha\text{sin}\delta_{i,3}}$. (S5b)

The kinematic variables can be replaced by the dihedral angles. Noting that , , , , , , Eqs. (S4), (S5a) and (S5b) become

, , (S6a)

, (S6b)

. (S6c)


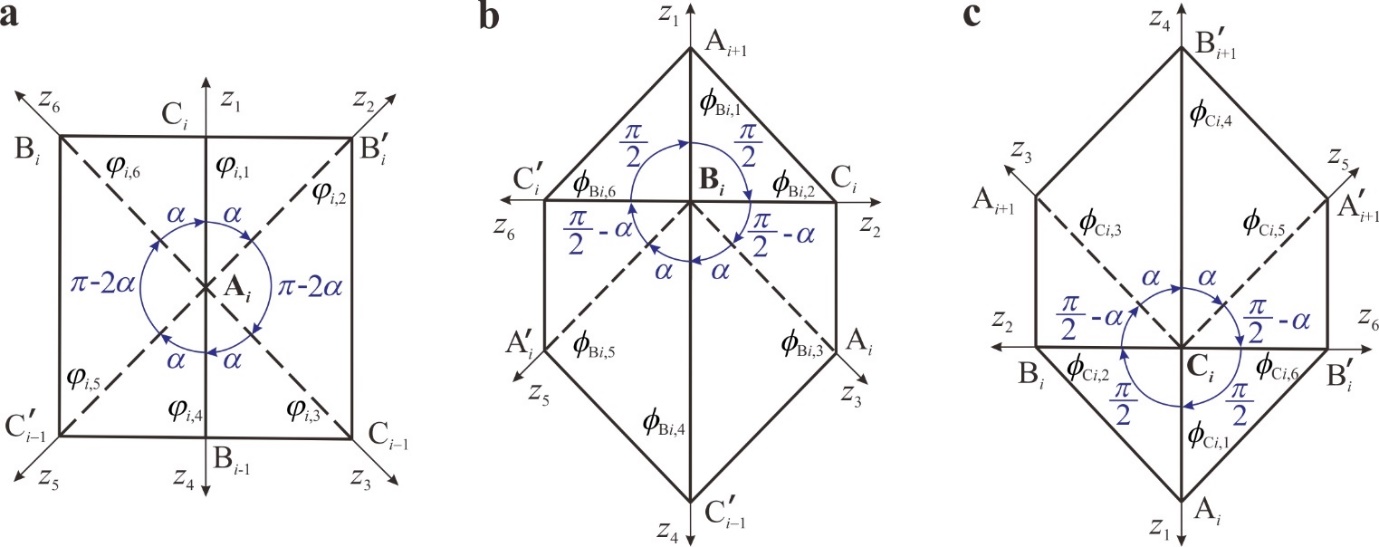


**Figure S2. a, b** and **c**, Spherical 6*R* linkage **A***_i_*, **B***_i_* and **C***_i_*, respectively.

For linkage **B***_i_* (Fig. S2**b**), its kinematic twists are $\alpha_{12}=\alpha_{61}=\pi/2,$ $\alpha_{23}=\alpha_{56} =\pi/2-\alpha,$ $\alpha_{34}=\alpha_{45}=\alpha.$ We have

$\omega_{\text{B}i\text{,}5}=\omega_{\text{B}i\text{,}3}$, $\omega_{\text{B}i\text{,}6}=\omega_{\text{B}i\text{,}2}$, (S7a)

$\text{tan}\frac{\omega_{\text{B}i\text{,}1}}{2}=\frac{\sin\alpha\cos\alpha(1-\text{cos}\omega_{\text{B}i\text{,}3})}{\sin\alpha(\text{sin}\omega_{\text{B}i\text{,}3}\text{cos}\omega_{\text{B}i\text{,}2}+\sin\alpha\text{sin}\omega_{\text{B}i\text{,}2}\text{cos}\omega_{\text{B}i\text{,}3})+\cos^{2}\alpha\text{sin}\omega_{\text{B}i\text{,}2}}$, (S7b)

$\text{tan}\frac{\omega_{\text{B}i\text{,}4}}{2}=\frac{\cos\alpha(\sin\alpha\text{cos}\omega_{\text{B}i\text{,}2}\text{cos}\omega_{\text{B}i\text{,}3}-\text{sin}\omega_{\text{B}i\text{,}2}\text{sin}\omega_{\text{B}i\text{,}3}-\sin\alpha\text{cos}\omega_{\text{B}i\text{,}2})}{\text{sin}\omega_{\text{B}i\text{,}2}\text{cos}\omega_{\text{B}i\text{,}3}+\sin\alpha\text{sin}\omega_{\text{B}i\text{,}3}\text{cos}\omega_{\text{B}i\text{,}2}}$. (S7c)

Again replace the kinematic variables with dihedral angels using , , , , , . Equations (S7a), (S7b) and (S7c) become

, , (S8a)

, (S8b)

. (S8c)

Similarly for linkage **C***_i_* (Fig. S2**c**), the following closure equations can be obtained,

, , (S9a)

, (S9b)

. (S9c)

Because each crease links two vertices, the dihedral angle on that crease is related to the motion of spherical linkages on both vertices, the compatibility between neighboring linkages **A***_i_*, **B***_i_* and **C***_i_* yields

, , , , . (S10)

These relationships hold for the entire waterbomb pattern.

**B. Motion of a tube with an odd number of rows**

**B.1 Kinematic analysis**

When *m* is odd, the equatorial row, named as row 0, exists. Linkage **A**_0_ not only has plane symmetry, it also has line symmetry, i.e., the lower half of the linkage is in rotational symmetry with the top half about an axis that passes through the vertex A_0_ and is perpendicular to the axis of the tube. The relationship between the dihedral angles of linkage **A**_0_ degenerates from Eq. (S6) to

, ,. (S11)

The projection of the row 0 onto the equatorial plane is shown in Fig. S3. Since

$\overline{\text{E}\text{E}'}=\overline{\text{B}_{0}\text{B}'_{0}}$, $\overline{\text{E}\text{E}'}=2a\sin\frac{\theta}{2}$ and ,

, (S12)

where $\theta$ is the folding angle between two largest triangular facets of the base with A_0_ as its central vertex. Moreover, from Fig. S3**b**, we have

$\frac{\phi_{\text{B0},4}}{2}+\frac{180^{\circ}}{n}=\frac{\varphi_{0,1}}{2}$. (S13)


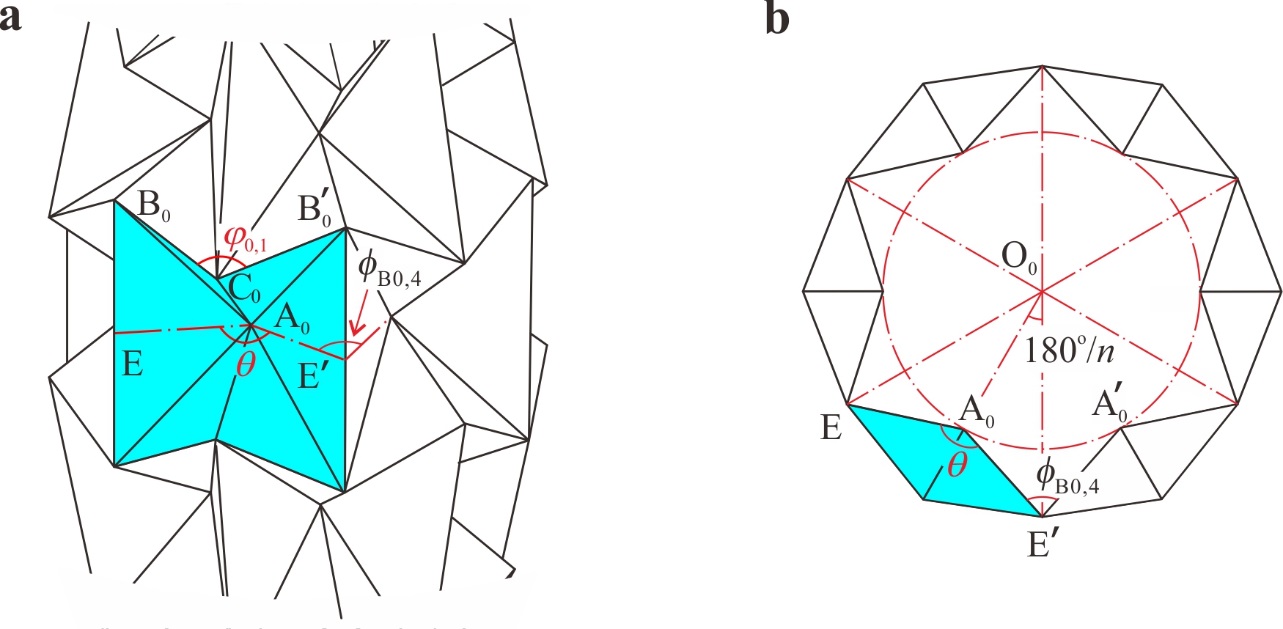


**Figure S3.** Projection of the waterbomb pattern when *m* is odd. **a**, front view; **b,** top view.

Therefore, the kinematic relationships of cylindrical waterbomb tessellation when *m* is odd presented by the dihedral angels are as follows.

For linkage **A**_0_,

, , ; (S14a)

for linkage **B**_0_,

$\phi_{\text{B0},4}=\varphi_{0,1}-\frac{2\pi}{n}$, ,

,,

;

(S14b)

for linkage **C**_0_,

, , ,

,

; (S14c)

for linkage **A**_1_,

, , ,

;

(S14d)

for linkage **B***_i_*,

, ,,

, ;

(S14e)

for linkage **C***_i_*,

, , ,

,

;

(S14f)

and for linkage **A***_i_*_+1_,

, , ,

,

(S14g)

where *i*= 1, 2, …, $\frac{m-3}{2}$.

Because the top and bottom halves of the tube have the same motion, hereafter only the equations for top half are given. Only  is needed to determine the motion of the entire waterbomb tube, which again demonstrates that the number of degree of freedom when the tube under radial and longitudinal symmetry is one.

The cylindrical coordinates of each vertex in the longitudinal strip as shown in Fig. 2(a) in the main text can be calculated as

, $z_{\text{B}i}=z_{\text{A}i}+\sqrt{(1+\cot^{2}\alpha)a^{2}-r_{\text{B}i}^{2}-r_{\text{A}i}^{2}+2r_{\text{A}i}r_{\text{B}i}\cos\frac{\pi}{n}}$;

, $z_{\text{C}i}=z_{\text{A}i}+\sqrt{a^{2}\cot^{2}\alpha-(r_{\text{C}i}-r_{\text{A}i})^{2}}$;

, $z_{\text{A0}}=0$, and $z_{\text{A(}i+1)}=z_{\text{B}i}+\sqrt{a^{2}\cot^{2}\alpha-(r_{\text{B}i}-r_{\text{A(}i+1)})^{2}}$, (S15)

where *i* = 0, 1, ..., $\frac{m-1}{2}$. And the overall length of the tube is

$L=2z_{\text{B(}(m-1)/2)}$. (S16)

Equations (S15) has been used to generate the plots given in Figs. 2(c), 3(b), and 3(c) in the main text in which the actuation angle is replaced by *θ* considering Eq. (S12).

There are two limit positions for the motion of the waterbomb tube. One is the most compact-folding cylindrical configuration when linkage **B**_0_ on the middle row is fully folded with , while the other one is the most deployed configuration when linkage **A**(*_m_*_-1)/2_ on row (*m*-1)/2 is fully deployed with . When , using Eq. (S13), we have

(S17)

On the other hand, when , by making the square root in the expression of in Eq. (S14g) zero, i.e.,

(S18)

is obtained. However, due to the highly nonlinear property of Eq. (S18), there are up to three solutions for . is the smallest of the solutions that are larger than $360^{\circ}/n$ whereas is the largest.

Because of Eq. (S12), the limits for are also limits for *θ*. In the main text, we present them as the limits for *θ*. Hence, within the range from $\theta_{\min}$ to $\theta_{\max}$, the folding of the waterbomb tube is rigid origami, and its kinematic paths, given by Eqs. (S14), can be plotted, see Figs. S4 and S5 for the tubes consisting of 3 and 7 rows, respectively.


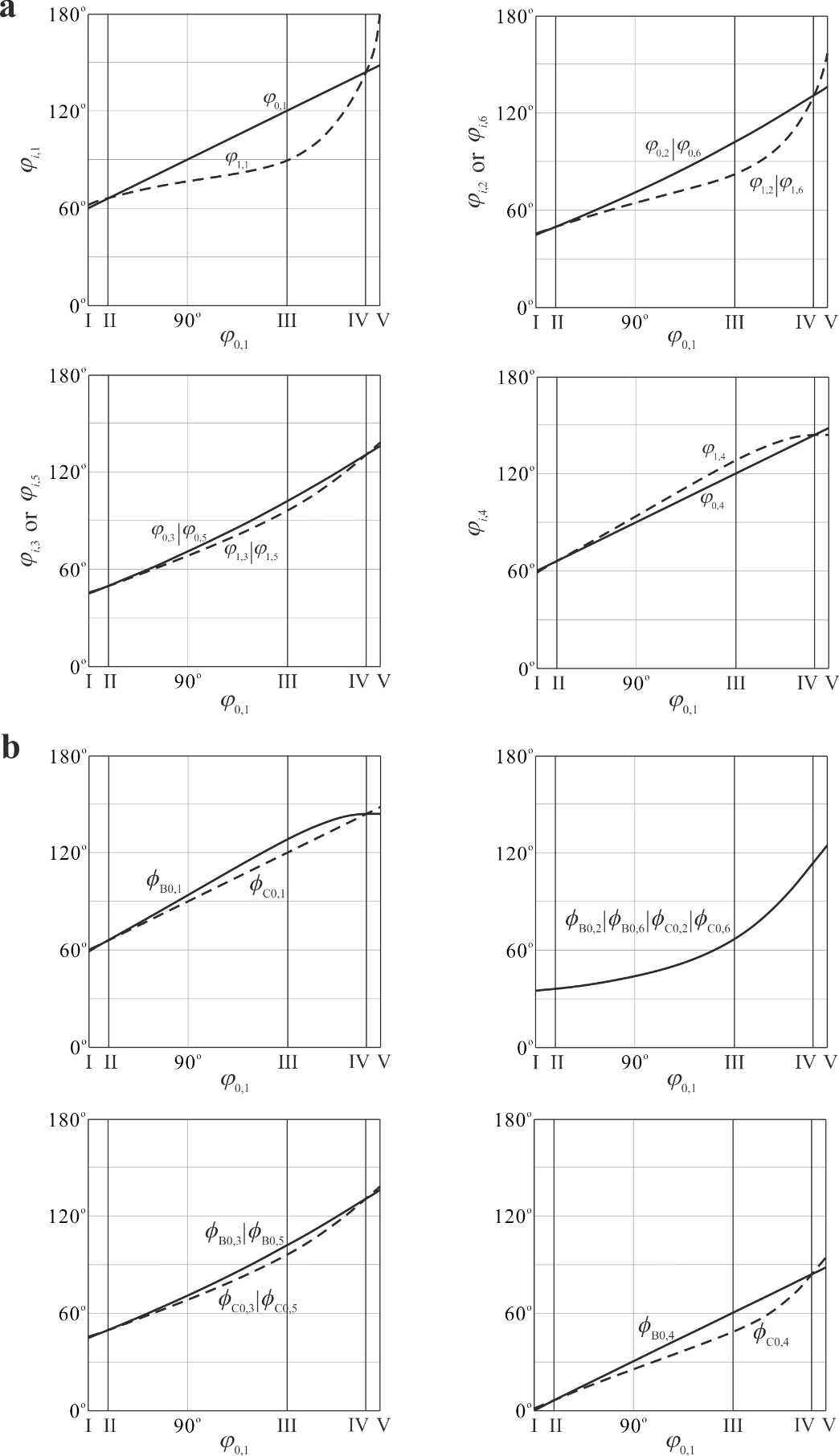


**Figure S4.** Kinematic paths of a waterbomb tube when $n=6$, $m=3$ and $\alpha=45^{\text{o}}$ for **a**, linkages **A**_0_ and **A**_1_; **b,** linkages **B**_0_ and **C**_0_. At I: , II: , III: , IV, , V: .

**
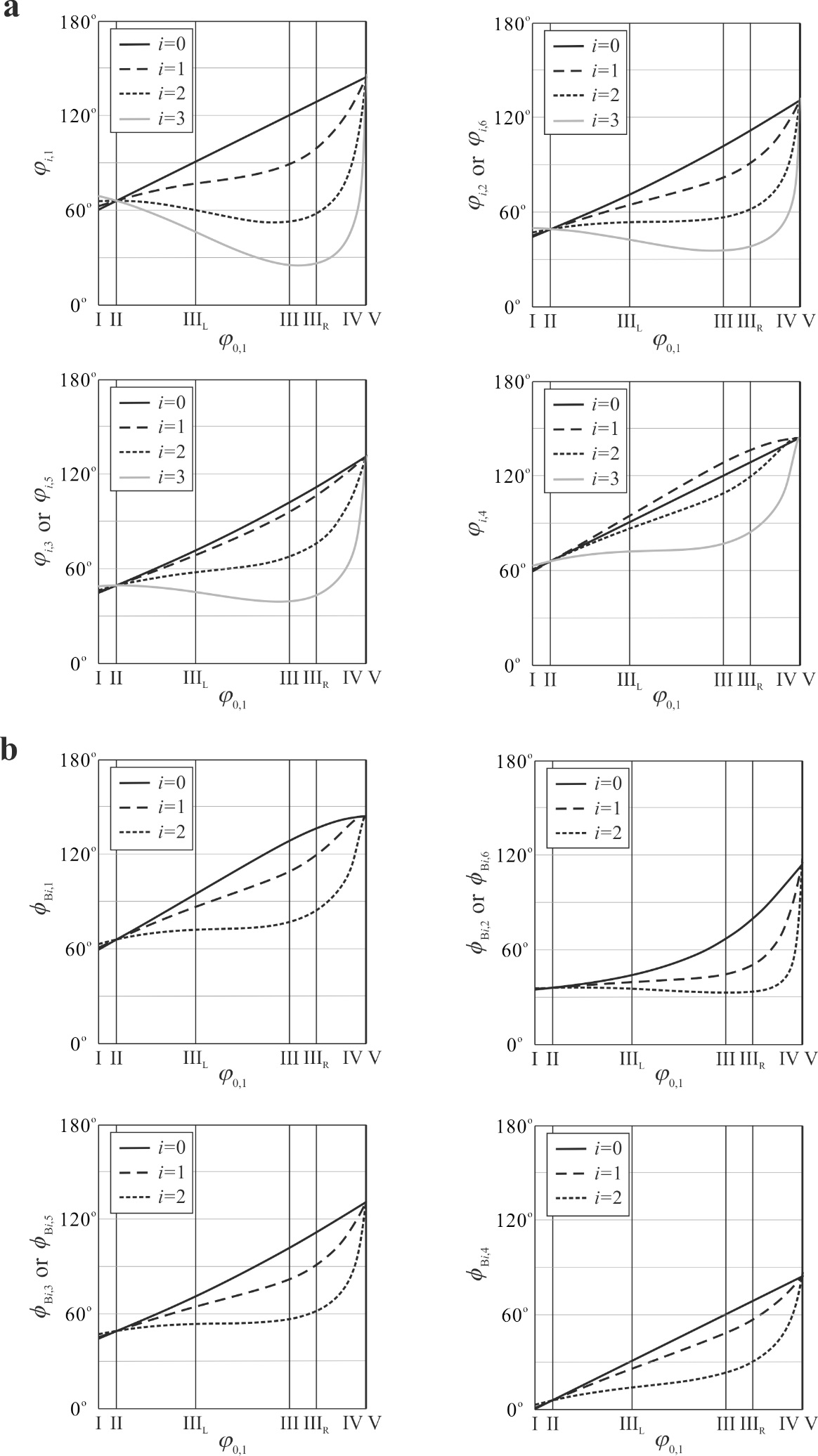
**


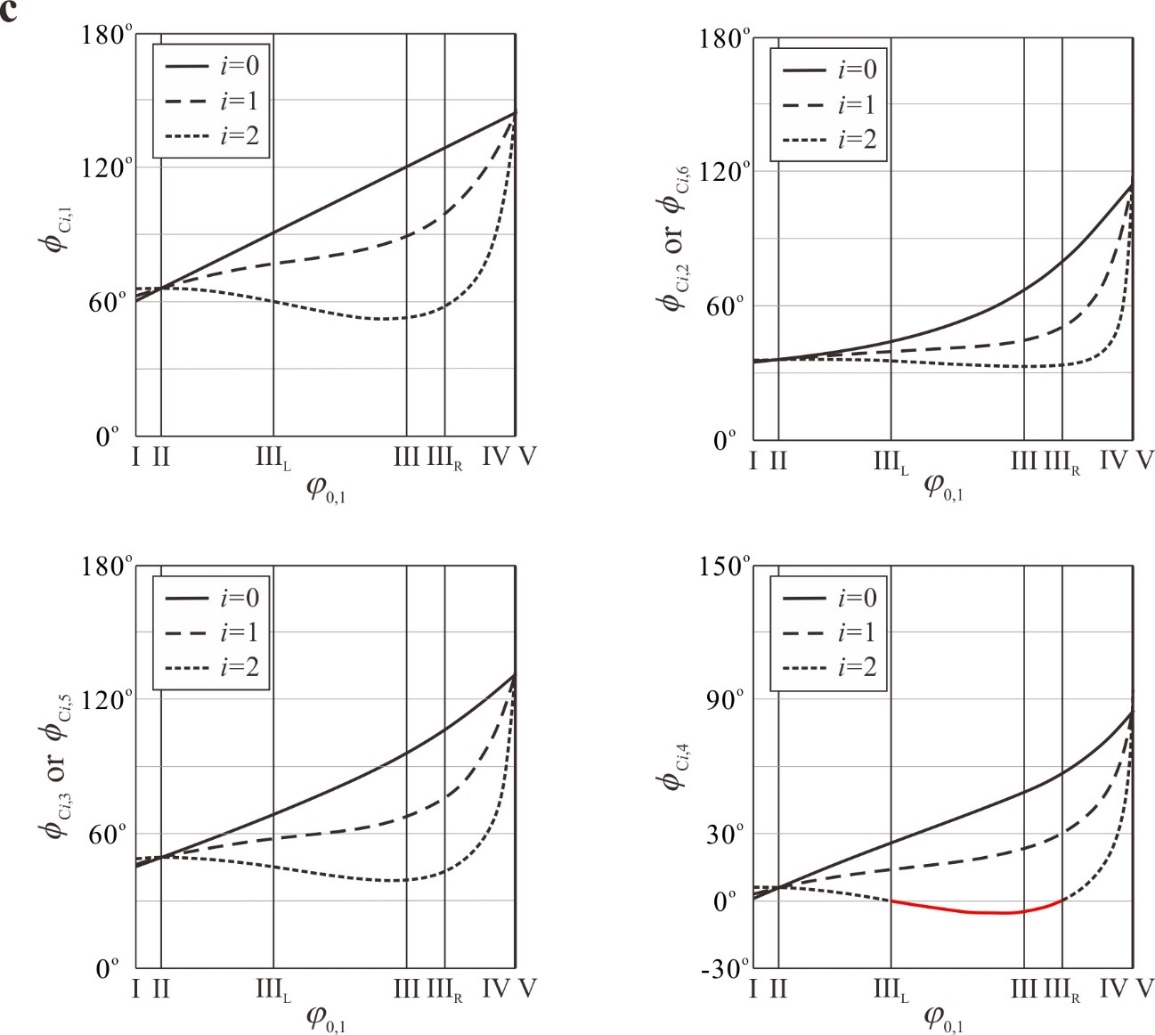


**Figure S5.** Kinematic paths of a waterbomb tube with $n=6$, $m=7$ and $\alpha=45^{\text{o}}$ for **a,** linkages **A***_i_*; **b**, linkages **B***_i_*; and **c**, for linkage **C***_i_*. At configurations I: ; II: ; III_L_: ; III: ; III_R_: ; IV: ; V:

**B.2 Uniform radius configurations of a tube**

When we fold the flat paper into the waterbomb tube, all the waterbomb bases are folded in an identical manner, i.e., all linkages **A** are in the same motion, so do linkages **B** and **C**. The relationship between the dihedral angles and can be calculated as

. (S19)

Once both Eqs. (S13) and (S19) are satisfied, the flat paper would fold to a waterbomb tube with a uniform radius. Take $n=6$ and $\alpha=45^{\text{o}}$ as an example. Substituting Eq. (S19) into Eq. (S13) gives

. (S20)

which yields two solutions for (or *θ*, due to Eq. (S12)): $65.88^{\text{o}}$ and $144^{\text{o}}$. These solutions correspond to configurations II and IV, respectively, given in the main paper. When a flat sheet of paper is folded according to the waterbomb pattern, the dihedral angle will be reduced from $180^{\text{o}}$. The first cylindrical shape reached will be the configuration IV as it has a larger radius than that of configuration II.

It should be pointed out that the existence of configuration of tube with uniform radius is solely decided by parameters $\alpha$ and *n*. It is not related to *m*. Moreover, the tube with a uniform radius can be obtained only when $n\geq5$ for $\alpha=45^{\text{o}}$. Figure S6 shows $\frac{\varphi_{0,1}}{2}-\frac{\phi_{\text{B0},4}}{2}$ *vs.* based on Eq. (S19). It can be seen that there is no intersection between line $\frac{180^{\text{o}}}{n}$ and curve $\frac{\varphi_{0,1}}{2}-\frac{\phi_{\text{B0},4}}{2}$ *vs.* when *n* < 5.

**B.3 Interferences at both ends of a tube**

Should a tube undergo a rigid origami motion, the radii in the cylindrical coordinates of any of the vertices must not be less than zero. Otherwise, interferences of facets would happen. Hence, on the verge of interference, one of $r_{\text{A}i}=0$, $i=0, 1, ..., \frac{m-1}{2}$. When one of $r_{\text{A}i}=0$, the corresponding

. (S21)


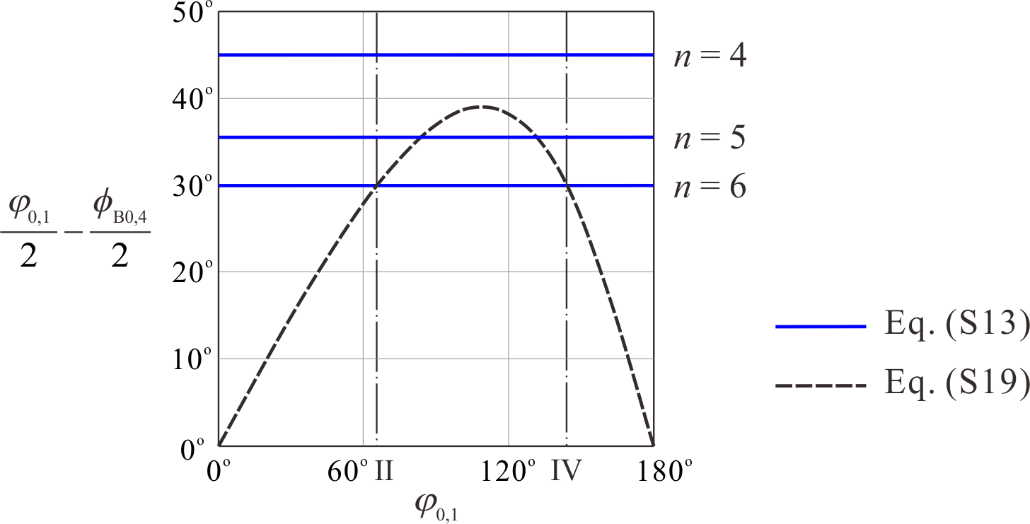


**Figure S6.** The configuration of tube with a uniform radius to be obtained by folding the flat origami pattern into the tube presenting with curve of $\frac{\varphi_{0,1}}{2}-\frac{\phi_{\text{B0},4}}{2}$ against when $\alpha=45^{\text{o}}$.

As the tube forms a pineapple shape between two uniform-radius configurations, it is apparent that the most likely set of vertices that would interfere with each other are the ones on the end row, i.e., $r_{\text{A[(}m\text{-1)/2]}}=0$. Therefore, considering Eq. (S21), . Using Eq. (S14e), , and thus, . Substituting it into Eq. (S14f) yields

. (S22a)

Replacing *i* with (*m*-3)/2 in Eq. (S14f) gives

(S22b)

In this equation, and are functions of because of Eq. (S14). So two unknowns, and at the verge of interference, can be obtained by solving simultaneously Eqs. (S22a) and (S22b).

Consider an example where $n=6$, $m=7$ and $\alpha=45^{\text{o}}$. Equation (S17) gives (or $\theta_{\min}=60^{\text{o}}$), whereas Eq. (S18) yields (or $\theta_{\max}=144.24^{\text{o}}$). The corresponding configurations of the tubes are named as configuration I and V, respectively. Simultaneous equations (S22a) and (S22b) lead to three solutions for : $41.20^{\text{o}}$, $90.72^{\text{o}}$ and $128.52^{\text{o}}$. Among them, two are located in the range between and , and so interference exists at , or $90.72^{\text{o}}\leq\theta\leq128.52^{\text{o}}$ within which ($\varphi_{\text{B}3,4}$) and $r_{\text{A3}}$ are negative as shown in Fig. S5**c** and Fig. 3(c) in the main text. The boundaries of that are named as configurations III_L_ and III_R_, with $\theta_{\text{III}_{\text{L}}}=90.72^{\text{o}}$ and $\theta_{\text{III}_{\text{R}}}=128.52^{\text{o}}$. Based on the above analysis, it can be concluded that the tube folds and expands as a mechanism within two distinct ranges: one between configurations I and III_L_, and the other between III_R_ and V. At configurations III_L_ and III_R_, the ends of the tube close, forming a concealed polyhedron, see configurations III_L_ and III_R_ in Fig. 3(a) in the main text. Moreover, the derivation above indicates that there is no bypass which satisfies rigid foldability between the two rigid motion ranges. Physically, the tube has to deform so as to move from one rigid motion range to the other. In other words, the tube works as a structure instead of a mechanism in this transition range.

The mechanism-structure-mechanism transition is only observed within a certain range of $\alpha$, which is also related to parameters *m* and *n*. $r_{\text{A3}}$ / *a* *vs*. *θ* and *α* diagram for a tube with *m* = 7, *n* = 6 is plotted in Fig. 3(d) in the main text. A number of observations can be made from this diagram. First, the mechanism-structure-mechanism transition only occurs when $44.63^{\text{o}}\leq\alpha\leq45.46^{\text{o}}$. Second, at $\alpha$ =$45.46^{\text{o}}$, $r_{\text{A3}}$ never becomes negative, and the rigid origami range for this tube is $60^{\text{o}}\leq\theta\leq143.93^{\text{o}}$. Moreover, closure of tube ends is observed at $\theta=115.27^{\text{o}}$ when $r_{\text{A3}}$ reaches zero. If $\alpha$ further increases, $r_{\text{A3}}/a$ *vs*. *θ* curve will move further upward with an increasing $\theta_{\min}$ and decreasing$\theta_{\max}$, and no closure of tube ends occurs. The rigid motion range shrinks with the increase of $\alpha$. Third, when $\alpha\leq44.63^{\text{o}}$, the mechanism-structure-mechanism transition also ceases to appear. The rigid motion range shrinks with a decrease in $\alpha$.

**C. Motion of a tube with an even number of rows**

For a waterbomb tube made of an even number of rows (i.e., *m* = even), there is no equatorial row that is plane- and line-symmetric. As a result, the equatorial plane slices through the middle points of the respective top and bottom edges of the bases on two rows immediately above and below the plane. To facilitate the derivation, we name the row below as row 0, and the row above row 1 (Fig. S7**a**).


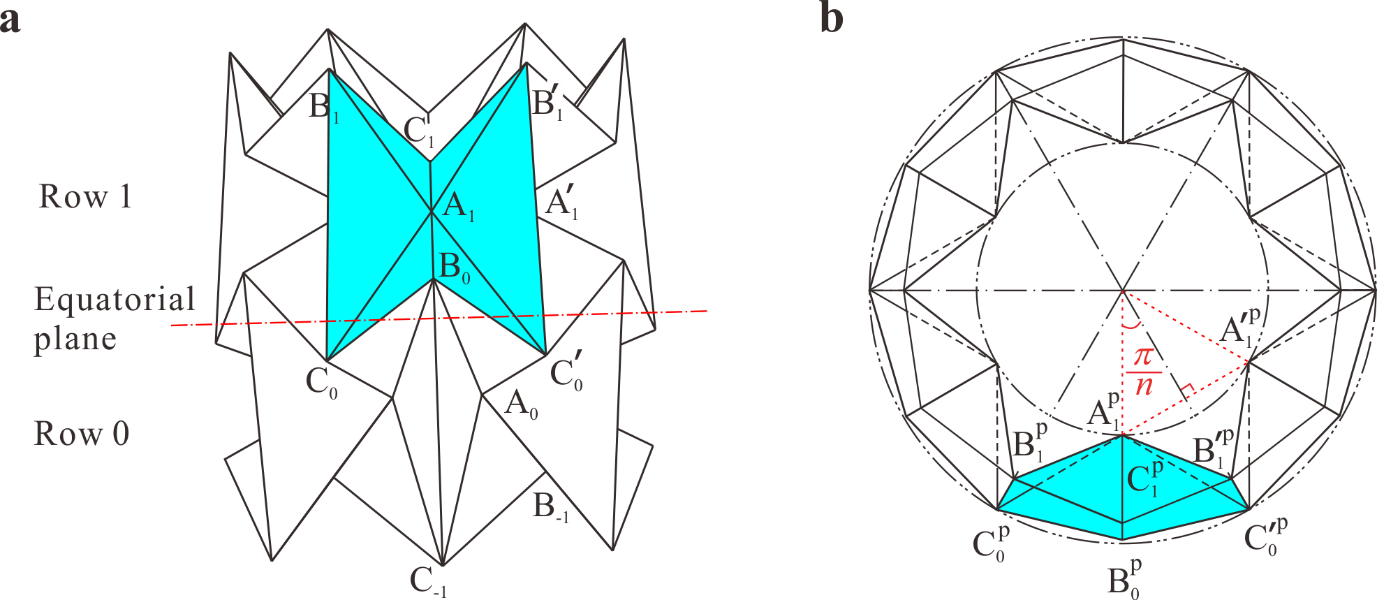


**Figure S7.** **a,** front view of row 1 in a tube where *m* is even; **b,** top view.

All the bases will have plane-symmetry. Equations (14e-g) still hold where *i* = 1, 2, …, $\frac{m}{2}-1$. Once the motion of rows 0 and 1 is determined, the motion of all rows can be obtained by Eqs. (S14e-g). Different from the odd-row case, the linkage **A**_0_ is no longer line- and plane-symmetric. As the top and bottom halves of the tube move in the same manner, linkages **B**_0_ and **C**_0_ are identical considering the half a base shift between adjacent rows, leading to

(S23)

The projection of row 1 onto the equatorial plane is presented in Fig. S7**b**. In order to complete a cylindrical tessellation, the following equation should be satisfied,

, (S24)

where $r_{\text{A1}}$ is given by Eq. (S15). Substituting Eq. (S15) into Eq. (S24) yields

, (S25)

which gives in terms of .

Due to the symmetry about the equatorial plane, creases $\text{A}_{0}\text{C}'_{0}$ and $\text{A}_{1}\text{B}_{0}$ in Fig. S7**a** have the same folding, i.e.,

. (S26)

Substituting Eq. (S26) into Eq. (S8), the following equations can be obtained

, (S27a)

, (S27b)

from which we can obtain and in terms of and .

Eliminating in Eqs. (S27a) and (S27b) gives the following equation,

(S28)

According to the theory of the quartic equation, the roots of the above equation can be obtained as

, (S29)

where

$U=\sin^{2}\alpha\cos^{2}\alpha(\tan^{2}\frac{\phi_{\text{B0},4}}{2}-\tan^{2}\frac{\varphi_{0,1}}{2}),$ $V=2\sin2\alpha\cos^{2}\alpha\tan\frac{\varphi_{0,1}}{2}(\tan^{2}\frac{\phi_{\text{B0},4}}{2}+1),$ $W=-2\cos^{2}\alpha[(2\cos2\alpha+\sin^{2}\alpha)\tan^{2}\frac{\phi_{\text{B0},4}}{2}+\sin^{2}\alpha\tan^{2}\frac{\varphi_{0,1}}{2}+2\cos^{2}\alpha]$,

and the choice of “+” or “–” is determined by the physical model.

Using Eqs. (S27a) and (S27b) again, we find

,

, ; (S30a)

, ,

; (S30b)

, , , ; (S30c)

, , , . (S30d)

It can be seen that only one free variable is needed to determine the motion of the waterbomb tube, meaning the number of degree of freedom of the tube in this case is again one. Note that is used as input here. The computational formula of the cylindrical coordinates of each vertex is as same as Eq. (S15). They are plotted in Fig. S8**b**, while the initial condition $z_{\text{A0}}=0$ is replaced by $z_{\text{B0}}\text{+}z_{\text{C0}}=0$ and *i* = 0, 1, 2, ..., $\frac{m}{2}$. The length of the tube is

$L=2z_{\text{B(}m/2)}$. (S31)

In order to find out the two limit positions: and as we did for the odd-row tube, one of the two critical conditions must hold: or . When , as well due to Eq. (S30c). It means that the two rows 0 and 1 are fully squeezed simultaneously, which is physically impossible. For , can be found from Eq. (S25). On the other hand, when , letting the square root of in Eq.  (S14g) be zero leads to

(S32)

Since and are functions of because of Eq. (S14), can now be obtained.

To acquire the corresponding when physical interference occurs, let , which is equivalent to from Eq. (S14e). We can recycle simultaneous equations in Eq. (S22) to obtain should row number $(m-3)/2$ in that equation be replaced by $m/2-1$. Now there are

(S33a)

(S33b)

from which we obtain on the verge of interference.

Take $n=6$, $m=8$ and $\alpha=45^{\text{o}}$ as an example. from Eq. (S25) and from Eq. (S32), and the corresponding configurations of the tube are named as configurations I and V, respectively. By solving simultaneous equations (S33a) and (S33b) with , two solutions of can be obtained: $81^{\text{o}}$ and $140.94^{\text{o}}$, indicating that interferences exist when . The boundaries are referred to as configurations III_L_ and III_R_.

Figures S8**a** and **b** show the kinematic relationships of the dihedral angles and radii of vertices with respect to , respectively. At configurations II and IV, corresponding to and $144^{\text{o}}$, respectively, the linkages **A**, **B** or **C** are all in the identical configurations, which are configurations corresponding to the tube with a uniform radius. These configurations are the same as those for a tube with odd rows. Between configurations III_L_ and III_R_, it is clear that both and $r_{\text{A4}}$ are negative. Hence, the mechanism-structure-mechanism transition also exists for tubes made from an even number of rows. For instance, by preserving *m* = 8, the mechanism-structure-mechanism transition occurs only when $44.66^{\circ}<\alpha<46.26^{\circ}$. If $\alpha\geq46.26^{\text{o}}$, $r_{\text{A4}}$ are positive and the rigid origami range of the tube decreases when $\alpha$ gets larger. When $\alpha\leq44.66^{\text{o}}$, the range of rigid origami range shrinks when $\alpha$ decreases.


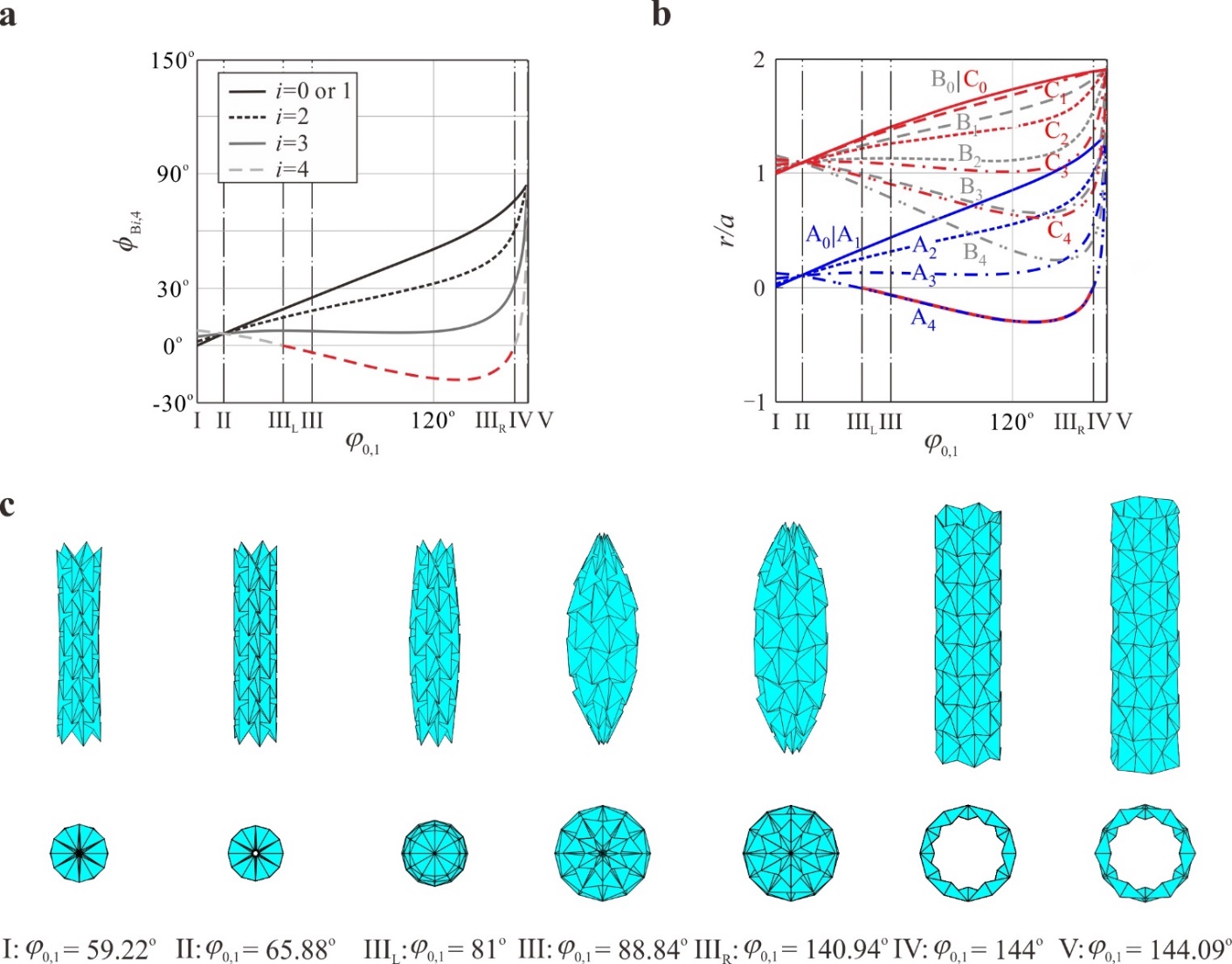


**Figure S8.** A waterbomb tube with *n*= 6, *m*= 8 and $\alpha=45^{\text{o}}$. **a,** the kinematic path *vs*. with between configurations III_L_ and III_R_ marked as red line; **b,** the radii of vertices A, B and C during the motion, where $r_{\text{A4}}<0$ between configurations III_L_ and III_R_ highlighted with blue line in red shadow; **c,** front and top views of the tube in the typical configurations I-V.

**S4. The mechanism-structure-mechanism transition**

Within the transition range, the assumption of rigid folding no longer applies, and material deformation on the facets should be taken into consideration. Therefore the results obtained from the kinematic analysis do not reflect the actual physical folding of the tube. To investigate the realistic mechanical behavior of the tube during the mechanism-structure-mechanism transition, a computational simulation approach using ABAQUS/Explicit was adopted.

A tube with *n*= 6, *m*= 7, and $\alpha=45^{\text{o}}$ was selected as an example for analysis. The tube was radially compressed from an initial folding angle $\theta$ of 130° to the final angle of 88°, covering the entire transition zone. Two cases, with and without facets intersection, were considered. When facets intersect, they were allowed to penetrate into each other, and the tube was expected to move in a way described by the previous kinematic analysis. If facet intersections were prohibited, self-contact of the tube was considered, and the tube had to deform for its facets to move.

The pattern parameter *a* and wall thickness *t* of the tube were chosen as 100mm and 1mm, respectively. Considering that physical creases had finite widths, strips of 1mm in width along each side of the creases formed the crease zone and the remaining parts formed the facet zone of the tube. Only one sixth of the tube was modeled because of symmetry. The tube was mainly meshed with four-node shell elements with reduced integration S4R, supplemented by a few triangular elements to avoid excessively small or distorted elements. Prescribed rigid body movements calculated from the kinematic analysis were assigned to the longitudinal edges on three rows to actuate the radial folding of the tube. Two linear elastic materials with different Young’s Modulus were assigned to the tube: 210 GPa for the facet zone, and 2.1 GPa for the crease zone. The density and Poisson’s ratio of both materials were 7800 kg/m^3^ and 0.3, respectively.

The radial folding of the tube without the consideration of the physical contact between the facets and vertices, named as mechanism mode, is shown in Fig. S9**a**. Note that although the tube is compressed with descending $\theta$, the results are still arranged in the order of ascending $\theta$ for consistency.

When facets are allowed to intersect, the numerical model (Fig. S9**a**) shrinks in a way identical to the kinematic model shown in Fig. 3(a). Radii of vertices A_3_, B_3_ and C_3_, plotted in blue in Fig. S9**c**, match those of the kinematic analysis. This confirms the findings of our kinematic analysis. It also indicates that the numerical model is also capable of simulating the rigid motion of the tube. If physical penetration of the facets were prohibited, the radial folding of the tube is shown in Fig. S9**b**. As predicted by the kinematic analysis, the vertices at the ends of the tube hit each other and deform during the process, which can be clearly observed from the configuration III. As a result, radii of vertices A_3_, B_3_ and C_3_ (plotted in black dashed lines in Fig. S9**c**), no longer match those of the kinematic model (grey lines in Fig. S9**c**), but all non-negative, which is named as the structural mode.

The behavior of the tube as a structure can best be illustrated by the difference in elastic strain energy stored in the facets between two modes, which demonstrates the net effect of the structural deformation in the tube. The strain energies of the facets, creases and the entire tube for the two modes are shown in Fig. S10**a** and **b**, respectively. In the mechanism mode most energy is stored in the creases, and the almost linear energy distribution is in correspondence with the rigid folding of the tube. A small amount of strain energy left in the facets is attributed to that they are not perfectly rigid. Note that the numerical simulation conducted here treats the tube as a continuum and the creases as having a finite width. This differs from the commonly used approach in which the creases act as elastic torsional springs, and the facets are rigid [37]. Hence, our approach more accurately reflects what happens in reality. In the structural mode, the strain energy in the facets (Fig. S10**b**) is nearly zero prior to configuration III_R_, exhibits a peak between configurations III_R_ and III_L_, and eventually falls close to zero after configuration III_L_. Recall that the radius of vertex A_3_ also shows a peak between configuration III_R_ and III_L_ in the kinematic modeling. This energy peak acts as an indicator of the level of deviation from rigid origami motion. The larger it is, the more deformation is required to enable the tube to move. The fact that it does not return to zero after configuration III_L_ can be attributed to that in the structural mode, some residual deformation remains in the tube after its ends open up again, which can be clearly seen at *θ* = 88° in Fig. S9**b**. This residual deformation slightly increases the overall energy level of the tube. Figure S10**b** also shows a peak in the strain energy of creases, which is again due to the continuum modeling approach which allows the creases to undergo deformation other than rotation along their axis. Comparisons of the strain energies of the facets, creases, and the whole tube between the two modes, are presented in Fig. S10**c** – **e**, respectively.


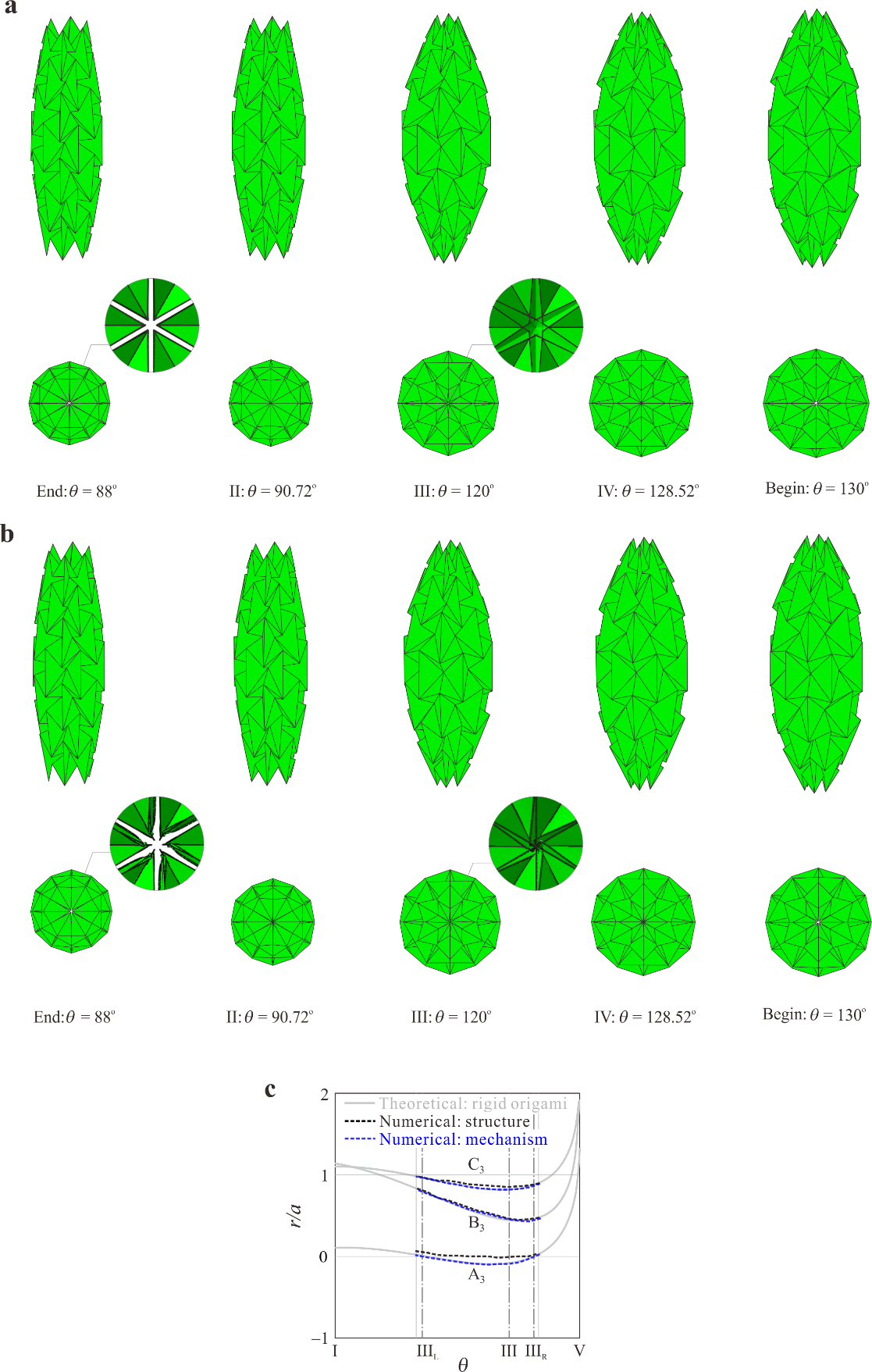


**Figure S9.** Numerical simulation of a tube with *n*= 6, *m*= 7 and *α*= 45°. Front and top views of the tube when facet penetrations were **a**, allowed, and **b,** prohibited. The enlarged figures show the deformation of the interfered vertices. **c,** Radii of vertices A_3_, B_3_ and C_3_ *vs*. *θ*.


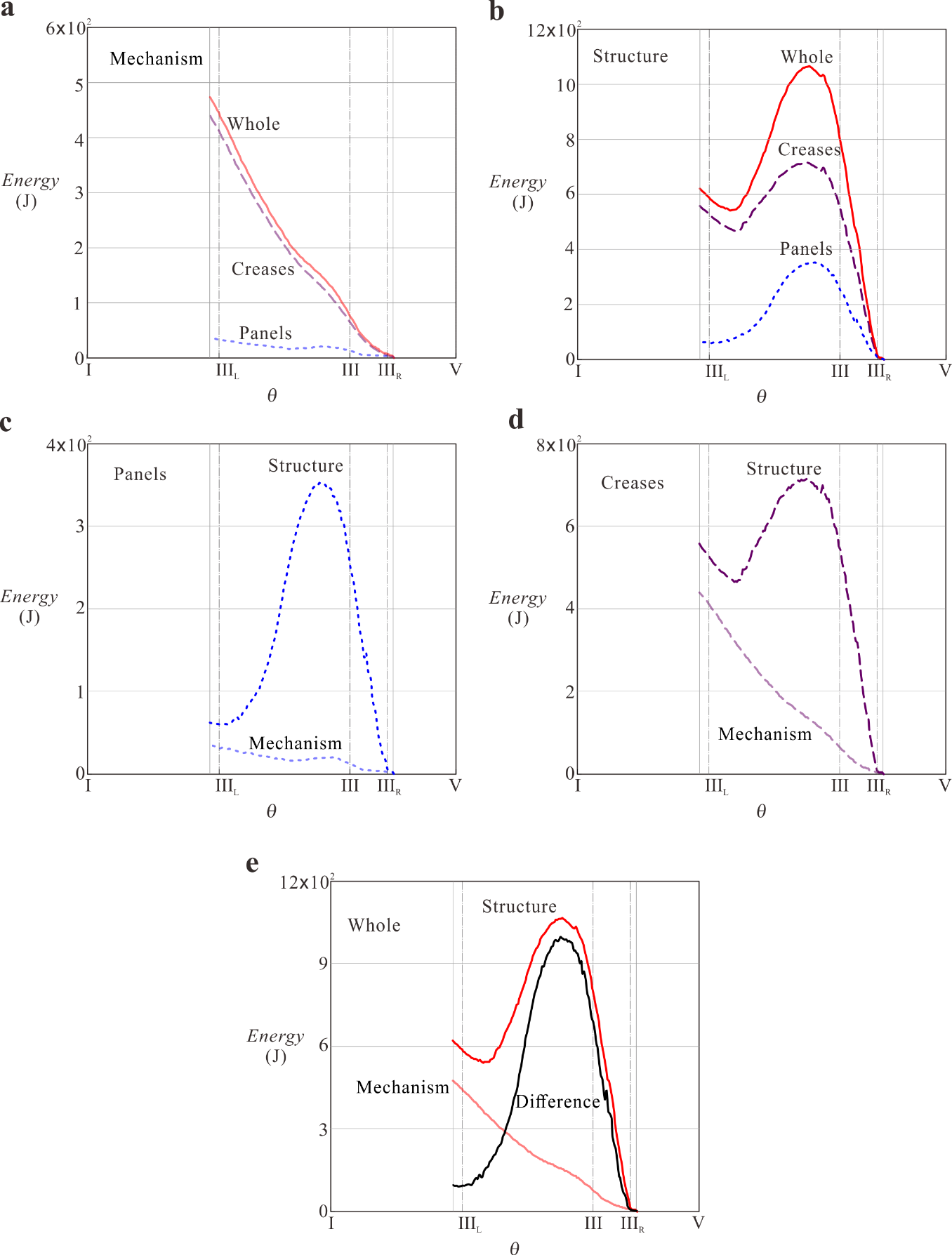


**Figure S10.** Strain energies of facets, creases, and the whole tube in **a**, the mechanism mode; and **b,** the structural mode. Comparison of energies on **c,** facets, **d,** creases, and **e,** the whole tube.

**References**

[36] J. Denavit and R. S. Hartenberg, J. Appl. Mech. **22**, 215 (1955).

[37] Z. Y. Wei, Z. V. Guo, L. Dudte, H. Y. Liang, and L. Mahadevan, Phys. Rev. Lett. **110**, 215501 (2013).
